# Protocol for the Optimizing Naloxone Dispensing in Pharmacies (ONDP) Online Continuing Education Program: A Randomized Controlled Trial

**DOI:** 10.3390/pharmacy10010024

**Published:** 2022-02-04

**Authors:** Ashley Cid, Alec Patten, Michael Beazely, Kelly Grindrod, Jennifer Yessis, Feng Chang

**Affiliations:** 1 School of Pharmacy, University of Waterloo, 10 Victoria St. S A, Kitchener, ON N2G 1C5, Canada; ashley.cid@uwaterloo.ca (A.C.); s2patten@uwaterloo.ca (A.P.); mike.beazely@uwaterloo.ca (M.B.); feng.chang@uwaterloo.ca (F.C.); 2 School of Public Health Sciences, University of Waterloo, 200 University Ave. W, Waterloo, ON N2L 3G1, Canada; jyessis@uwaterloo.ca

**Keywords:** naloxone, harm reduction, community pharmacy, continuing education, behaviour change, stigma, program evaluation

## Abstract

The number of opioid-related deaths in Canada has steadily increased since 2016 and the COVID-19 pandemic has worsened this trend. Naloxone has been pivotal for reducing opioid-related harms and death, and pharmacists play a crucial role in ensuring the supply of naloxone to Canadians through community pharmacies. However, naloxone dispensing by pharmacists is not optimal; in fact, in Ontario, only 50% of pharmacists offer naloxone, despite national guidelines that pharmacists should offer naloxone to everyone with an opioid prescription. When asked why pharmacists do not proactively offer naloxone, recent research has identified that pharmacists need continuing education to boost confidence and knowledge on how to start conversations with patients. The study involves a delayed start, double-blind randomized controlled trial, for Canadian licensed pharmacists and pharmacy technicians. The goals of the program are to increase Canadian pharmacy professional’s knowledge, confidence, and motivation to proactively offer naloxone, as well as to decrease stigma associated with naloxone. The program incorporates behaviour change techniques from the Theoretical Domains Framework and the Theory of Planned Behaviour. The intervention program includes three modules that focus on improving pharmacists’ communication skills by teaching them how to proactively offer naloxone, while the control group will complete a reading assignment on the naloxone consensus guidelines. The program will involve a process and outcome evaluation in addition to a contribution analysis. This program is important for breaking down previously identified barriers and knowledge gaps for why pharmacists currently do not proactively offer naloxone. This study will provide important new information about what behaviour change techniques are successful in improving confidence and motivation in the pharmacy profession and in an online environment. Findings from this study can be used to produce a national naloxone education program that can also be implemented into current pharmacy school curriculum.

## 1. Introduction

### 1.1. The Opioid Crisis in Canada

The opioid crisis is growing every year, which is related to an alarming number of cases of opioid-induced respiratory depression (OIRD) due to both prescription and unregulated opioids. In Canada, over 17,000 opioid-related deaths have occurred between January 2016 and June 2020 [1]. These deaths are impacting national life expectancy rates [2], and opioids have now surpassed motor vehicle accidents as the leading cause of accidental death [3]. Between January and June 2020, and the start of the COVID-19 pandemic, 97% of these opioid-related deaths were accidental and could have been prevented [1]. Due to the social distancing restrictions imposed to lessen the effects of the COVID-19 pandemic, unintended harm has been put on individuals who consume prescription or unregulated opioids [4]. In the first fifteen weeks of the COVID-19 pandemic, there was a 38.2% increase in opioid-related deaths reported, in comparison to the fifteen weeks before the start of the pandemic [4].

Prescription opioids such as codeine, morphine, oxycodone, and hydromorphone are frequently used for treating pain-one of the most common reasons for acquiring healthcare in Canada [5]. Approximately 1 in 8 (~4.7 million) people in Canada, were prescribed an opioid in 2018, which represents a vast number of people at risk for OIRD and death [6]. In addition to prescription opioids, diverted prescription opioids, unregulated heroin, and an increased prevalence in unregulated fentanyls are contributing to opioid overdoses and fatalities [7,8]. During the COVID-19 pandemic, fentanyl and its analogues are considered major drivers in the opioid crisis with 75% of opioid-related deaths involving fentanyl and 85% of opioid-related deaths involving unregulated opioids [1]. Due to the drastic rise in opioid-related deaths, a highly accessible, safe, and effective life-saving solution was needed; this solution is also known as naloxone [5].

### 1.2. Take-Home Naloxone in Canada

In response to the opioid crisis, naloxone, an opioid receptor antagonist was removed from the prescription drug list by Health Canada in 2016 [5]. This change allowed naloxone to be distributed to the public without a prescription [5]. The use of naloxone as a temporary antidote to OIRD has been a pillar for harm reduction in Canada, as it has facilitated the distribution of naloxone to not only injection drug users or high-dose prescription opioid patients, but to family members, friends, bystanders, and service providers [9]. In Canada, two formulations of naloxone are available, intramuscular (IM) and intranasal (IN). IM naloxone is publicly funded in all provinces and territories, but IN naloxone is publicly funded in only Ontario, Quebec, and the Northwest Territories [10]. Access to these kits is highly variable as the programs are managed at the provincial level [9,10]. For example, one can receive a free take-home naloxone (THN) kit at over 1000 distribution sites in Alberta, but there are over 3000 sites in Ontario [10,11]. This variation in public access to THN creates barriers for obtaining this life-saving medication.

### 1.3. Pharmacy-Based Take-Home Naloxone Programs

As one of the most accessible healthcare providers, pharmacists are ideally positioned to play a role in the opioid crisis by providing naloxone and education to patients and their family who may be at risk of OIRD or bystanders of a potential OIRD situation [12]. Pharmacy-based THN programs allow for the provision of naloxone to eligible patients by registered pharmacists as per the naloxone consensus guidelines [5]. Eligible patients include those at risk of an opioid overdose, a friend, family member or a potential bystander of someone at risk for an opioid overdose. Although naloxone kits are theoretically available in all community pharmacies based on its removal from the prescription drug list, many variations exist in community pharmacies with respect to THN access, knowledge, and willingness to obtain or train how to use naloxone [5,13,14]. Most THN programs through pharmacies are not mandatory and there is a considerable variability in the number of pharmacies who stock and/or dispense naloxone [13].

A recent scoping review identified multiple sources that studied the availability of naloxone and its dispensing patterns within pharmacies. It found that few pharmacists ever offer or dispense naloxone, despite stocking it in their pharmacies [13,15,16,17,18,19,20]. For example, a survey of 284 community pharmacists in Indiana found that over half (58.1%) of pharmacies stocked naloxone, but only 23.6% of pharmacists ever dispensed it at the pharmacy [21]. In addition, multiple sources found that naloxone is less likely to be available in districts with high rates of opioid overdose deaths, minority neighbourhoods and areas with lower household income [20,21,22,23].

The disparity in accessing naloxone was also worse for rural pharmacies and independent pharmacies; these were less likely to stock and dispense naloxone compared to urban and chain community pharmacies [15,18,19,20,24,25,26,27,28,29]. In addition, just over half of all pharmacies in Ontario dispensed naloxone in 2018, and a third of the kits dispensed were from only 1% of all pharmacies in large urban centres [15]. When a naloxone kit is dispensed, a study examining pharmacy dispensing data found that the most common reason for dispensing naloxone was the patient’s request, and 89.5% of patients indicated that it was their first naloxone kit [26]. A survey examining the dispensing habits of pharmacists during the COVID-19 pandemic found that one of the main barriers for not dispensing naloxone during the pandemic was the lack of patients asking for a naloxone kit [30]. The survey also found that the pharmacists that were proactively offering kits and making adaptations to offer naloxone kits were doing so over the phone or by delivery, to work around less face-to-face interactions between the pharmacists and patients during the COVID-19 pandemic [30]. Therefore, although many patients are not benefiting from the THN program and many pharmacists are not proactively offering naloxone, there is a need for increased and equitable naloxone distribution through community pharmacy THN programs and there are new and innovative approaches to better THN dispensing.

### 1.4. Barriers and Facilitators for Optimal Naloxone Distribution

In the most recently published scoping review of THN programs, one of the top reported barriers for optimal naloxone distribution was stigma from both the patient and pharmacist perspectives [14]. From the patient perspective, they reported not feeling comfortable asking for a naloxone kit because they were afraid of future consequences, such as a potential change in the pharmacist’s attitude towards the patient if they ask for naloxone, or a fear of being labelled by the pharmacist as an “addict” for having a history of naloxone on their patient profile [14]. Pharmacists reported being afraid of offending a patient when offering them a naloxone kit. For example, some pharmacists mentioned that they were uncomfortable introducing the subject of naloxone with patients they felt were at risk of opioid overdose and stated that they were unsure how to introduce the topic of opioid overdose and naloxone, as they did not want their patients to feel judged [14].

As a means for reducing stigma, the recent Canadian national consensus guidelines for naloxone prescribing by pharmacists suggest offering a naloxone kit to everyone with an opioid prescription and was mentioned in a study by a patient who suggested having store policies for pharmacists universally offering naloxone [5]. Another solution that was found by the scoping review was to increase advertising and marketing of naloxone inside pharmacies, such as having naloxone pamphlets available for patients to present to a pharmacist to reduce the need for verbally asking for a naloxone kit and increase the privacy of the patient [14]. Many pharmacists still hold negative beliefs about naloxone fueled by misinformation, such that it leads to riskier opioid use, or it allows people who use opioids illicitly to avoid seeking substance use treatment [14]. Other reported barriers included concerns about incorporating naloxone efficiently within the pharmacy workflow, and fears about justifying the need for naloxone when a patient refuses an offer for a naloxone kit [14]. Often patients hold the incorrect belief that they are not at risk for OIRD if they have been taking chronic prescription opioids, and pharmacists require communication tools to respond to these views [14].

Common facilitators for dispensing naloxone include education and training for both pharmacists and patients and comfort in discussing naloxone, such as initiating conversations with patients [14]. A study that implemented a communication pocket-card for pharmacists and provided techniques for starting conversations with patients resulted in a four-fold increase in naloxone dispensing [31]. Studies have also described recommendations made by pharmacists for future educational topics to address knowledge gaps. These topics include methods for naloxone counselling and identifying patients who would benefit from naloxone [32].

## 2. Experimental Design

### 2.1. Study Objectives and Design

Based on the recent scoping review by Cid et al. and the recent Canadian national consensus guidelines for naloxone prescribing by pharmacists, there is a need for a continuing education program for Canadian pharmacists to optimize naloxone dispensing [5,14]. Based on the fact that stigma is one of the top barriers preventing optimal naloxone dispensing, an intervention is needed to combat opioid and naloxone related stigma for pharmacy professionals, in order to increase naloxone distribution [5,14] Such a program is integral to the education of pharmacists by breaking down barriers and providing necessary information for them to effectively take on this role within their communities. This manuscript describes a proposal for the Optimizing Naloxone Dispensing in Pharmacies (ONDP) continuing education (CE) program, the results of this study will be published upon completion of the methods described in this manuscript. The objectives of the ONDP CE program are to increase pharmacy professional’s knowledge about naloxone and increase their confidence and motivation to proactively offer naloxone. Additionally, this program aims to decrease stigma associated with naloxone and opioids and increase the amount of naloxone kits being dispensed in community pharmacies.

The ONDP program will occur online. Computer-based education has the advantage of being flexible, adaptable to different learning preferences, anonymous, applicable to diverse clinical contexts, and unbound by geography and classroom/instructor availability [33]. It can be delivered asynchronously, allowing the learner to choose the time and place to learn [33]. Computer-based education also appears to improve knowledge, but systematic reviews have highlighted the need for high-quality data on its effects on behaviour [34,35]. This CE program will be designed using the Theory of Planned Behaviour (TPB) and the Theoretical Domains Framework (TDF) along with their corresponding behaviour change techniques to increase confidence and motivation to proactively offer naloxone [33,36].

The ONDP program will be conducted as a two-arm, delayed-start, double-blind, randomized controlled trial. The trial will be conducted in accordance with the CONSORT guidelines and has received ethics clearance by the University of Waterloo research and ethics board (ORE: 43699). Eligible participants include licensed Canadian pharmacy technicians, and pharmacists who are classified as “Part A,” meaning that they are actively involved in direct patient care. Participants must be working within a community pharmacy practice for at least 1 year, and the pharmacy must dispense a minimum of 30 prescriptions per day. This eligibility criteria were chosen to ensure that the participants have enough experience working in community pharmacy, and that the pharmacy is dispensing enough prescriptions to be able to practice the content being taught in the ONDP program. Pharmacy technicians are included in this study due to their ability to improve workflow-based barriers pharmacists have previously described in other studies [14]. For example, pharmacy technicians will learn from the CE program how to ask if a patient would like a naloxone kit upon receiving an opioid prescription at prescription intake, or they can ask a patient over the phone if they would like a naloxone kit when a patient calls to refill an opioid prescription. Based on the findings from the scoping review by Cid et al., it will be important to recruit both urban and rural working pharmacy professionals, as it was shown that rural pharmacies are less likely to stock or offer naloxone [14]. Demographic data for each participant will be collected at the beginning of the program during the “Introduction Survey,” where the population centre of each participant’s workplace will be asked [37].

### 2.2. Sample Size

A sample size of 100 participants was calculated based on prior studies using the “Opening Minds Stigma Scale for Health care Providers” (OMS-HC). This scale was developed and validated to measure mental health related stigma in healthcare professionals and has been found to have good internal consistency using the Cronbach’s alpha coefficient (a = 0.74–0.79) and has been shown in other randomized controlled trials to detect positive changes in various anti-stigma interventions [38]. Based on the trial by Beaulieu et al., 111 physicians were assigned to either intervention or control groups, and the OMS-HC scale was administered [39]. Their calculations indicated that 50 physicians would be required in each group, under the assumption, 80% power would be achieved to detect a between-group difference in mean scores of 3 points on the OMS-HC scale with an alpha value of 5%. Clinically meaningful change was defined as a change of 3 points. Similarly, another randomized controlled trial by Fernandez et al., randomly assigned 102 medical students to either arm, where the OMS-HC scale was administered pre-, post-treatment and at 1-month follow-up [40]. Based on these findings, we will enroll a minimum of 100 pharmacy professionals (either pharmacists or pharmacy technicians) into the ONDP study, as this sample size has previously been an effective measure when using the OMS-HC scale, as a measure of stigma in healthcare professionals. We structured the sample size around stigma measurement, due to it being a primary objective, and the main reason behind low pharmacy naloxone dispensing numbers.

### 2.3. Recruitment and Incentives

Recruitment will occur mainly via social media (Twitter and Facebook). Those registrants will then be e-mailed by Dr. Cid to notify them about the research study. Once participants finish the online program and the 3-month follow-up survey, they will be entered into a draw for one of six Amazon $50 gift cards. The participants who decide to also complete the one-on-one interviews after the 3-month follow-up survey, will receive $50 in payment for their time. All participants will have the benefit of continuing their professional development for free, while also learning about topics that are not widely accessible or regularly taught.

## 3. Materials/Equipment

Both the intervention and control programs are hosted on Thinkific.com (accessed on 30 December 2021), a learning management system for hosting course content. All course material was prepared using Articulate Storyline (accessed on 30 December 2021), and the external program resources were designed by Adrian Poon, a graphic design, and motions artist [41]. Both arms of the ONDP program, including all the program resources, were peer-reviewed by 3 licensed pharmacists. All course content was edited and updated upon the reviewers’ comments. The ONDP intervention program also underwent beta-testing via the fourth-year pharmacy student cohort at the University of Waterloo. As part of their naloxone education in their professional practice course, all 119 students completed the intervention portion of the program and provided comments about their experience with the content.

### 3.1. The Intervention Program

The intervention program will consist of three separate modules; it will take approximately three hours to finish the entire program. Module 1 of the CE program will focus on practical ways pharmacists can improve their communication skills to proactively offer naloxone. This module will build upon the barriers, facilitators and knowledge gaps that were previously mentioned such as strategies for starting conversations with patients [14]. The goal of this module is to provide practical solutions for how pharmacists can overcome the barriers that prevent optimal naloxone dispensing. This module will include access to infographics, pocket cards and pharmacy display resources which can be readily used in pharmacies to help optimize naloxone dispensing. Pocket cards were identified in the scoping review by Cid et al. as a valuable tool to reinforce concepts such as patient populations who should get a naloxone kit and includes common rebuttals to a patient’s refusal of a naloxone kit [14,35]. Pharmacy display resources include pamphlets that patients can access and use to present to a pharmacist or pharmacy technician without having to ask for naloxone and have others waiting in the pharmacy area hear them. This was also determined in the scoping review as a tool to help reduce the stigma of asking for a naloxone kit [14,42,43]. This module will also include approaches for including naloxone strategically within the pharmacy workflow, how to optimize the use of pharmacy team members, how to build confidence about starting conversations and how to avoid stigmatizing language when offering a naloxone kit.

Module 2 will focus on Frequently Asked Questions (FAQs) about naloxone such as: whether THN programs reduce OIRD fatalities, does naloxone increase or promote opioid use, what is “Good Samaritan” legislation, are there pharmacokinetic differences between IN and IM naloxone, does naloxone break down if not stored properly, do unregulated fentanyls mean that standard naloxone kits are insufficient, can you suffer a fentanyl OIRD via accidental exposure and if an OIRD is successfully reversed are there long-term negative outcomes. The answers to these FAQs were compiled through a series of literature reviews and can be found in the special series of the Canadian Pharmacists Journal. They are aimed at correcting misinformation and knowledge gaps related to naloxone [44,45,46]. This module will provide pharmacists with the tools needed to answer FAQs from patients and colleagues and correct any inaccurate perceptions pharmacists may have about naloxone.

Module 3 will be built based on a survey of Ontario pharmacists and pharmacy technicians on their habits of offering naloxone during the COVID-19 pandemic and how these adaptations can be used going forward to overcome barriers to naloxone access [30]. The survey determined how naloxone dispensing patterns have changed since the start of the COVID-19 pandemic in comparison to before the pandemic started [30]. The survey also gathered what adaptations pharmacists have made to dispense naloxone kits now that most people are staying at home and what barriers and facilitators exist for dispensing naloxone during the pandemic [30]. The results from the survey were used to create a module that optimizes naloxone dispensing with COVID-19 restrictions in mind and overcomes barriers for naloxone dispensing that have been created because of the COVID-19 pandemic. The goal of Module 3 will be to provide some direction for pharmacists on how naloxone can still be proactively offered during the COVID-19 pandemic [30].

### 3.2. The Control Program

The control program will follow the same pre- and post-evaluation processes as the intervention program, discussed later in this manuscript. Instead of three separate online modules, the participants of the control program will read a publication on Thinkific. This publication is published in a national Canadian journal and is entitled, “Canadian national consensus guidelines for naloxone prescribing by pharmacists”. This publication serves as the standard training approach for offering naloxone in current pharmacy practice and was used in the development of the intervention arm.

### 3.3. Theories of Behaviour Change

The Theory of Planned Behaviour (TPB) and the Theoretical Domains Framework (TDF) are the behaviour change theories that are incorporated throughout the ONDP program [33,36]. The use of these theories allows for a targeted program that will allow for knowledge, confidence, and motivation to be gained according to the TPB and TDF. The TPB and the TDF were discovered through a review of similarly designed programs in the literature to be effective and practical theories to implement in the ONDP program to emphasize factors that influence behaviour change [36,47]. The TPB involves structuring the ONDP program to build confidence and motivation to strengthen the likelihood of changing a particular behaviour, in this case, proactively offering naloxone [33]. This theory promotes behaviour change because if a participant believes a behaviour to be easier to achieve, they are more likely to change compared to an individual with the same intentions but a perception that the behaviour is more difficult to achieve [33]. The TDF provides a guide for researchers to implement behaviour change theory into educational programs [35]. It’s composed of twelve domains that were developed from a systematic review of 33 behaviour change theories [36]. The focus of this framework is to address barriers that contribute to less successful behaviour change, as well as consider how an individual’s personality can affect their adoption of new behaviours [36]. Four of the 12 domains will be incorporated into the program design: social influence, beliefs about capabilities, skills, and behavioural regulation [36].

### 3.4. Implementing Behaviour Change Techniques: Theory of Planned Behaviour

The TPB involves changing behaviour by shaping an individual’s intentions and perceived behavioural control [33]. To shape the intentions of pharmacists, the following techniques will be used in the online program: information on others’ approval, information about health consequences, goal setting and personalized learning [33,35]. For example, information on others approval will be implemented through videos and quotations of other pharmacists discussing how they proactively offer naloxone or why the dispensing of naloxone is an important practice [36].

Personalized learning was found to be an effective technique at improving clinicians’ intentions to change their behaviour [48]. To improve intentions, the participants will be asked to reflect on their current knowledge of naloxone to enable them to create personalized learning goals of what they want to complete when working on the module. These learning goals will be incorporated into each of the modules to allow participants to reflect and work towards their goal of completion throughout the program. The participants will be asked if they completed their learning goals by the end of the online program and will be asked to set goals based on what they have learned in their daily pharmacy practice [48]. In addition, reflection questions and statements will be added throughout the modules to allow pharmacists to reflect on their past and current understanding of how to offer naloxone, to improve their intake of new knowledge and boost confidence in their perceived ability to proactively offer naloxone [49,50]. In a three month follow up survey, the participants will be asked to reflect on whether they achieved their goals.

### 3.5. Implementing Behaviour Change Techniques: Theoretical Domains Framework

#### 3.5.1. Social Influence

The social influence domain focuses on the influence pharmacists’ peers and colleagues have on their current behaviour as well as behaviour change [36]. Social norms, also known as social influence, can help to shape the participants’ intention to perform a behaviour [33]. Based on a study of pharmacists and the TDF, it was found that pharmacists perceive themselves as role models for their pharmacy colleagues [36]. Social influence can be implemented through videos of pharmacists modelling positive behaviour change, videos of pharmacy colleagues discussing their approval of the goal behaviour as well as information on how pharmacists can positively impact their pharmacy through becoming a role model [36]. Modelling will also be used throughout the online program as this technique provides a demonstration of behaviour to participants to develop their skills and see other pharmacists engaging in the behaviour [48]. Modelling will be implemented using quotations displayed in flashcard and video format.

#### 3.5.2. Beliefs about Capabilities

The beliefs about capabilities domain of the TDF also connects with the TPB where perceived behavioural control has an impact on the likelihood of behaviour change [33]. Techniques that can be used to increase pharmacists’ likelihood of behaviour change include behavioural rehearsal, graded tasks, and credible sources [36]. The technique of behavioural rehearsal and graded tasks will be implemented through scenario-based questions, graded tests as well as the modelling videos of other pharmacists [36]. The technique of credible sources will be incorporated through the module content where access to further resources and references will be hyperlinked [36]. Participants will also have access to infographics and posters which can be downloaded and used in the pharmacy.

#### 3.5.3. Skills

The theoretical domain of skills is focused on developing pharmacists’ knowledge and skills in performing specific tasks in pharmacy practice, inadequate training and a lack of skill can be detrimental to patients [36]. The skills domain has been proposed to be effective in online learning for pharmacists when it is framed through the demonstration of behaviour of others, behavioural rehearsal through case-based quizzes, graded tasks, instructions on how to perform the behaviours (infographics, videos), and interactive decision-making scenarios [36]. All these techniques will be implemented into the online program.

#### 3.5.4. Behavioural Regulation

The theoretical domain of behavioural regulation allows for the incorporation of reflection about behaviour in the intervention to encourage a more desired or positive behaviour [36]. The technique that will be incorporated into the naloxone program is self-monitoring of behaviour [36]. Questions to spark self-reflection and self-feedback relating to the participants’ new behaviour or behavioural goals will be incorporated throughout the naloxone intervention to meet the technique of self-monitoring of behaviour [36]. By incorporating self-reflection, the participant has a chance to think about their goal behaviour, and regulating their own behaviour within their pharmacy practice, which can motivate behaviour change.

### 3.6. Measuring Stigma

The “Opening Minds Stigma Scale for Health care Providers” (OMS-HC) was selected to measure stigma related to opioids in the ONDP program. The scale includes 15 questions that use a 5-point Likert scale to quantify pharmacists’ stigma towards mental illness [51]. The scale measures three subtypes of stigma which are: attitudes, disclosure, and social distance [51]. The attitudes subscale measures what health care providers think towards patients with mental illness. For example, a pharmacist may believe their patients with mental illnesses are liars or lazier than those that do not have a mental illness [51]. The disclosure subscale measures a health care providers’ willingness to seek out help or communicate to others that they have a mental illness [51]. The third subscale, social distance, measures a healthcare providers’ level of comfort or discomfort working or living close to a person that has a managed mental illness [51]. These subscales are used to categorize the 15 questions within the stigma scale and help to quantify a pharmacists’ level of stigma towards mental illness.

For the purposes of the ONDP program, the OMS-HC scale was adapted to measure opioid-related stigma, rather than mental illness. To select the wording for the adapted OMS-HC scale, a survey was conducted with 119 pharmacy students who were randomized into 4 subsections. Each subsection used different wording to describe opioid consumption: opioid addiction, opioid use disorder, opioid misuse/use disorder and opioid dependency. Preliminary results demonstrated that “opioid addiction” was the most stigmatizing word choice to help measure stigma (unpublished data). The OMS-HC scale was adapted to use opioid addiction in the place of mental illness in each of the 15 questions. Participants’ scores on the OMS-HC scale will show the overall level of stigma towards opioid addiction with a higher score demonstrating the participant has a higher level of stigma [38]. The introduction, conclusion and 3-month follow-up surveys will be compared to see if there is a drop in the participants’ score after completing the program, as this will indicate a drop in stigma.

The OMS-HC scale was selected due to its reliability and testing across multiple research papers. In an article testing the efficacy of their intervention of mental illness, the OMS-HC scale was found to have an internal reliability of a = 0.79 with the three subscales having internal consistencies of a = 0.68 for attitudes, a = 0.67 for disclosure and a = 0.68 for social distance [51]. Similar internal reliabilities were found in an examination of the OMS-HC scale with the overall scale having a Cronbach’s alpha of 0.74 to 0.79 [38]. A good internal consistency with a Cronbach’s alpha of around 0.7 was also documented when the OMS-HC scale was adapted to measure stigma towards border line personality disorder [52,53]. Social desirability bias was tested with the OMS-HC scale and was weakly correlated with the stigma scale [54]. The OMS-HC scale was chosen to be adapted for this program because the scale was crafted specifically to measure health care providers’ stigma and was rigorously tested through content validation, stakeholder consultations and health care provider focus groups [54].

## 4. Detailed Procedure

### 4.1. Evaluation Design

The ONDP program will be evaluated using a mixed methods process and outcome evaluation, with a contribution analysis approach [55,56]. The process evaluation will be used to identify the areas for future program improvement, and areas that participants felt were effective for learning, while the outcome evaluation will be focused in identifying if pharmacists report a decrease in stigma related to opioids, and an increase in knowledge, confidence, and motivation to proactively offer naloxone, and an increase in the number of naloxone kits dispensed [56]. The contribution analysis approach will be helpful in determining if the online continuing education program contributes to the program outcomes [55]. The evaluation will include the following components: a pre- and post- knowledge test (MCQ) to measure knowledge change. An introduction, conclusion and a 3-month follow-up survey to measure confidence, motivation, and numbers of naloxone kits dispensed. Finally, stigma will be measured by including the OMS-HC survey in the introduction, conclusion, and follow-up surveys. After the 3-month follow-up survey, all participants will be invited to participate in one-on-one interviews to expand on their responses from the 3-month follow-up survey.

The introduction survey will be used to collect demographic data, baseline levels of naloxone distribution, confidence, ability, and motivation levels in proactively offering naloxone, as well as a rating of how important community outreach is and how motivated they are to participate in it. The conclusion survey will follow the post knowledge test and will collect information about how to improve the program such as suggestions for content addition/removal, changes in program formatting, and what strategies were motivating for changing current behaviour. The conclusion survey will also collect self-reported confidence, motivation, and ability levels in proactively offering naloxone. The 3-month follow-up survey will collect information about the number of naloxone kits being dispensed, goal setting, and the use of the module resources, as well as confidence and motivation levels. The survey will also ask about specific strategies that pharmacists have implemented into their workplace to promote naloxone dispensing. The knowledge tests and surveys will be analyzed using descriptive statistics in Microsoft Excel, and IBM SPSS software will be used to conduct t-tests, odds ratios, and chi-square tests to measure changes in knowledge, confidence, and motivation pre- and post-program. Any qualitative responses from the surveys, and the one-on-one interviews will be thematically coded by A.C. and a research assistant.

The contribution analysis approach was chosen because it is a theory-based approach to assessing the contribution of an intervention to the observed results [57,58,59]. A contribution analysis uses a theory of change to connect the intervention with the expected impacts, allows you to examine the activities of the intervention, the underlying external factors and the assumptions of the intervention that produce the given outcomes [57,58,59]. A contribution analysis will allow us to conclude with confidence that the ONDP program is contributing to the observed results [57,58,59]. The contribution analysis will follow the six steps developed by Mayne and will involve the creation of a contribution analysis team which is recommended by Riley et al., to allow for a team-based approach to the evaluation [58].

The Reach, Effectiveness, Adoption, Implementation, Maintenance (RE-AIM) framework will also be applied to the program evaluation. The RE-AIM model is an evidence-based approach for evaluating the population impact of a public health intervention [60]. Reach is the representation of program participants from the target group; a good representation will translate to a better case for generalization in the real world [60]. The effectiveness domain will involve determining the efficacy in the program’s ability to improve pharmacist’s naloxone dispensing practices [60]. The adoption domain involves the number of participants who adopted the practices learned within the program, and whether it was adopted by other colleagues [60]. The implementation domain determines to what extent the program was delivered as intended [60]. The program intends to be consistently delivered due to its online nature. The evaluation will involve measuring the number of participants who complete all the program components including the follow-up survey. The maintenance domain involves a measure of the extent to which the program’s content was sustained over time [60]. This will be analyzed by the number of participants who continue to implement what they learned in the program three months after completing the program.

### 4.2. Program Procedure

**Figure 1:**
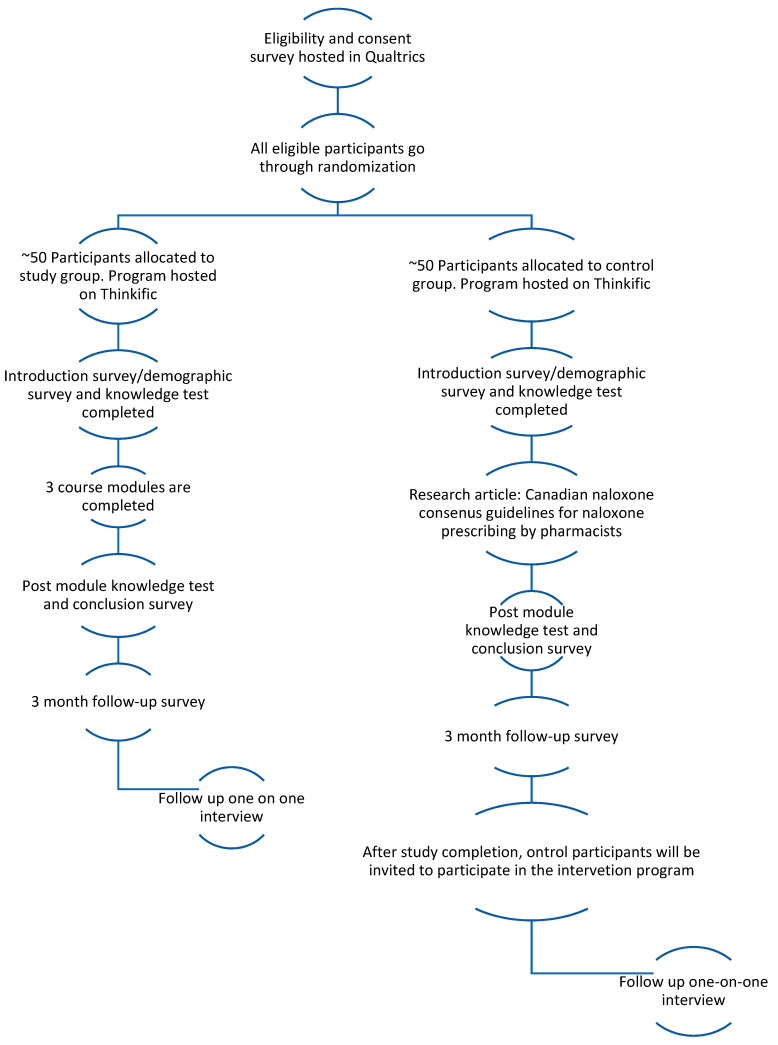
Flow Diagram of Program Procedure [5].

Participants will be invited to participate in the study via e-mail or social media (Facebook or Twitter). The invitation to participate will explain the purpose of the study and will provide a link to the information and consent letter hosted in Qualtrics survey software. The participants can provide consent to participate in the study via a radio button, then they will be forwarded to the eligibility quiz in Qualtrics. If they are deemed eligible based on the quiz, the participants will be forwarded to a separate page in Qualtrics to provide their e-mail so that A.P. may add them to the Thinkific platform (Figure 1). Participants will be randomly allocated by A.P. to either the intervention group (ONDP 3 module program), or the control group (ONDP reading assignment), which both occur as separate courses in Thinkific (Figure 1). The randomization will occur via a random number table. The participants in the control group will be notified by e-mail that they must complete the assignment reading program first, prior to gaining access to the ONDP program (ONDP 3 module program). Both arms have 14 days to complete their respective programs (either the assignment reading program or the ONDP 3 module program). For the control group, they will have an additional 14 days to complete the ONDP 3 module program upon completion of the assignment reading program. Reminder e-mails to complete the program content will be sent via e-mail after 7 days, and again after 11 days of the initial invitation to improve response rates. All participants will be instructed via the information and consent letter that they have 14 days to complete their assigned program. No development or refinement of the courses will be performed on the platform or modules while conducting the study. A.C. will be blinded to the participant allocation and will only receive the downloaded and de-identified data from A.P. upon the end of the program study period (28 days) and again upon participant completion of the 3-month follow-up survey.

In the Thinkific control group course, entitled: “Optimizing Naloxone Dispensing in Pharmacies Assignment”, the participants will complete a pre-knowledge test (multiple choice) and introduction survey (Figure 1). Next, they will be directed to read a publication on Thinkific entitled “Canadian national consensus guidelines for naloxone prescribing by pharmacists,” (total of 5 pages). This publication is published in a Canadian journal and serves as the standard training approach being used for all pharmacists in practice. Once the participants have finished reviewing this publication, they will be instructed to complete the post-knowledge test and the conclusion survey in Thinkific (Figure 1). At the end of the survey, the participant will be thanked for completing the assignment, and that they will be added to the ONDP program at the end of the 14-day assignment program period.

In the Thinkific intervention group course entitled: “Optimizing Naloxone Dispensing in Pharmacies Online Continuing Education Program,” the participants will complete a pre-knowledge test (multiple choice), which not an exact replica of the control group test, 18 out of 36 test questions are the same, the remaining questions are new. Then, they will complete an introduction survey to gather baseline information about their demographics, naloxone dispensing habits and levels of stigma (OMS-HC scale) (Figure 1). Next, they will be directed to complete 3 online modules that cover how to proactively offer naloxone (3 h). At the end of module 3, the participants will be directed to complete the post-knowledge test, and the conclusion survey (Figure 1). Then, the participant will be reminded that they will receive an e-mail in 3 months to remind them to complete the follow-up survey.

The 3-month follow-up survey will be hosted in a separate course in Thinkific from the intervention and control group courses (Figure 1). All participants from the intervention and control group arms will be automatically enrolled after 3 months, when the reminder/invitation e-mail to participate in the follow-up is sent out. The participants will be told via the invitation e-mail that they have 2 weeks to complete the survey, with a reminder after the first 7 days after the initial invitation. The final question in the survey will ask if the participants would like to be contacted for participation in a one-on-one phone interview to gather more insight to the answers.

A.P will assign a unique study identifier to each participant of the trial prior to analysis of the data. A.P will be unable to add the study identifier into Thinkific as each participant is associated with an e-mail to be able to access the content in the platform, and to be able to send reminder e-mails. Once the results are downloaded from the platform, they will be password protected, and de-identified prior to data analysis occurring with A.C. Once all analysis has been completed on the Thinkific program, the Thinkific course will be deleted including all study participants. With this program deletion, the study researchers will request an information deletion from the Thinkific database which will delete and remove all student information entirely from their database.

For the one-on-one interviews, participants will be e-mailed based on whether they agreed to be contacted in the follow-up survey for an interview. The interview will take 30 min of their time and be conducted over the phone. The target population for the one-on-one interviews will be participants who reported in their follow-up survey that they are not proactively offering naloxone, have low motivation/confidence to offer it and/or they still report high levels of opioid-related stigma based on the OMS-HC scale. This population is preferred to allow for an increased understanding of where the program can improve and identify further barriers for low naloxone dispensing rates. Participants who completed both the assignment and the ONDP program will be asked to compare their experiences with the control and intervention arm programs. The participants unique study ID for the program will be added to the interview recording prior to transcription and data analysis.

## 5. Expected Results

The authors expect that pharmacists who complete the ONDP program will increase their naloxone dispensing rates and increase their knowledge, confidence, and motivation in proactively offering naloxone. The authors also expect to see a decrease in opioid-related stigma among pharmacy professionals. The primary outcome of this program is to determine if there is a decrease in opioid-related stigma among pharmacy professionals after completing the ONDP program. The secondary outcome will be to determine if pharmacists’ have an increase in confidence and motivation in proactively offering naloxone. The third outcome will be to determine if naloxone dispensing rates and knowledge about naloxone increases among pharmacy professionals.

### 5.1. Limitations

Some potential limitations of this study include the need for participants to ensure they have a stable internet connection for participation. We do not suspect this to be a major limitation as all pharmacies operate with a computer system that has access to the internet, and if pharmacists choose to complete the program at home, Statistics Canada reports that 94% of Canadians have internet access at home [55]. The second limitation involves potential difficulty recruiting participants during the COVID-19 pandemic where many pharmacies are currently focused on vaccination efforts. Since the program includes education about how to incorporate naloxone dispensing during COVID-19, the program can be accessed at any time and place, including a phone or tablet, and the negative impact the pandemic has had on the opioid crisis being a current topic of interest, we chose to still carry out the study as planned. The final limitation involves the timeline of the study. Since the study is funded for only a short period of time, a longer follow-up could not be chosen. A three-month follow-up would still allow for data collection, analysis, and dissemination by the end of the study grant. It is unclear if a 3-month follow-up is long enough to effectively measure behaviour change.

### 5.2. Implications and Future Research

This program has the potential to allow for further understanding on how to effectively motivate pharmacists to proactively offer and dispense naloxone. Upon further development or improvement of the program based on feedback from this trial, this program has the potential to become a standardized continuing education program that is offered to all Canadian pharmacists with the goal of optimizing naloxone distribution. This study will provide new information about what behaviour change techniques are successful in the pharmacy profession and in an online environment. Findings from this study can be used to design other continuing education programs with different topics. This program has the potential to educate pharmacists in hard-to-reach areas of Canada such as isolated rural or northern populations which do not regularly have access to resources or in-person training. Future research can expand on the study of this program by allowing for a longer follow-up to measure behaviour change. It may also be useful to study the impact of the opioid crisis and offering naloxone by including it on prescriptions with opioids, and to study the impact of universal offering of naloxone by pharmacists.

### 5.3. Conclusions

This report described the design and evaluation of a national online CE program for pharmacists with the focus of optimizing naloxone distribution. This study is crucial for breaking down previously identified barriers and knowledge gaps for why pharmacists currently do not proactively offer naloxone, or why they do not offer naloxone at all. Once pharmacists feel that they have the knowledge, confidence, and ability to be able to offer naloxone kits, they can continue to be crucial frontline workers who can make a difference in reducing the impacts of the opioid crisis.

## Data Availability

Not applicable.
